# The development of fluorescent protein tracing vectors for multicolor imaging of clinically isolated *Staphylococcus aureus*

**DOI:** 10.1038/s41598-017-02930-7

**Published:** 2017-06-06

**Authors:** Fuminori Kato, Motoki Nakamura, Motoyuki Sugai

**Affiliations:** Department of Bacteriology, Hiroshima University Graduate School of Biomedical and Health Sciences, Hiroshima, 734-8551 Japan

## Abstract

Recent advances in fluorescent protein technology provide a wide variety of biological imaging applications; however current tools for bio-imaging in the Gram-positive bacterium *Staphylococcus aureus* has necessitated further developments for fluorescence intensity and for a multicolor palette of fluorescent proteins. To enhance the expression of multicolor fluorescent proteins in clinical *S*. *aureus* strains, we developed new fluorescent protein expression vectors, containing the *blaZ*/*sodp* promoter consisting of the β-lactamase gene (*blaZ*) promoter and the ribosome binding site (RBS) of superoxide dismutase gene (*sod*). We found *S*. *aureus*-adapted GFP (GFP_sa_) driven by the *blaZ*/*sodp* promoter was highly expressed in the *S*. *aureus* laboratory strain RN4220, but not in the clinical strains, MW2 and N315, harboring the endogenous *blaI* gene, a repressor of the *blaZ* gene promoter. We therefore constructed a constitutively induced *blaZ*/*sodp* promoter (*blaZ*/*sodp*(Con)) by introducing substitution mutations into the BlaI binding motif, and this modification allowed enhanced expression of the multicolor GFP variants (GFP_sa_, EGFP, mEmerald, Citrine, Cerulean, and BFP) as well as codon-optimized reef coral fluorescent proteins (mCherry and AmCyan) in the *S*. *aureus* clinical strains. These new fluorescent probes provide new tools to enhance expression of multicolor fluorescent proteins and facilitate clear visualization of clinical *S*. *aureus* strains.

## Introduction

Fluorescent proteins are widely used as biological markers that enable visualization of subcellular protein localization, gene expressions, protein-protein interactions, and *in vivo* monitoring of bacterial infection^1–3^. Currently available fluorescent proteins are primarily derived from either the green fluorescent protein (GFP) originating in the jellyfish *Aequorea victoria* ^4, 5^, or reef coral fluorescent proteins (RCFP) derived from *Discosoma* sp^5–7^. The wild type *A*. *victoria* GFP exhibits poor fluorescent brightness in *Escherichia coli* and mammalian cell lines; and many of the wild type GFP have a strong tendency to be expressed as an insoluble protein, showing cytotoxicity in *E*. *coli* ^8, 9^. To date, extensive studies have reported numerous types of *A*. *victoria* GFP variants that provide significant improvements in brightness, protein solubility, stability, pH-sensitivity, and yield^4, 5, 9^. Further, numerous variants of GFP and RCFP with distinct colors have been engineered using a combination of random mutagenesis and directed evolution. This has enabled co-visualization of several proteins in a single cell, selective identification of particular cells in co-culture systems, and detection of protein-protein interactions based on a measurement of fluorescence resonance energy transfer (FRET)^1–5^.

*Staphylococcus aureus* is a low-GC Gram-positive bacterium that causes a variety of diseases, e.g., abscess, bullous impetigo, toxic shock syndrome, pneumonia, sepsis, and food poisoning. Multidrug-resistant strains such as methicillin-resistant *S*. *aureus* (MRSA) cause severe hospital-acquired infections such as pneumonia and sepsis; and the resistance makes the treatment increasingly difficult. The development of molecular genetic tools including gene deletion, controllable gene expression, and bio-imaging is essential for our understanding of the mechanisms underlying the pathogenesis of *S*. *aureus* infections^10, 11^. Currently, for bio-imaging numerous GFP and RCFP variants are commercially available from many distributors; however, these FPs were not optimized for less common bacterial strains (e.g., clinical strains of *S*. *aureus*). Bio-imaging tools based on fluorescent proteins have also been widely used in the studies of *S*. *aureus* to visualize subcellular proteins or for biofilm formation as well as to establish a *S*. *aureus* infection model using fluorescent proteins, in which optimization of codon usage and replacement of the region surrounding the ribosome binding sequence (RBS) have been reported to enhance the expression of the fluorescent proteins in *S*. *aureus* ^12–16^. However, the current methods often entail several limitations as to color palette and its brightness and therefore necessitated the development of new fluorescent vectors that can efficiently enhance fluorescence intensity and the multicolor palette in *S*. *aureus* strains. Many proteins are generally difficult to highly express in heterologous host organisms. In *E*. *coli*, extensive research has established many different types of promoters and a great number of engineered laboratory strains for heterologous protein production^17^. However, a well-established method for the heterologous protein expression is still lacking in *S*. *aureus*, much less clinically isolated strains. Previous studies have focused on the β-lactamase gene (*blaZ*) promoter *P*_*blaZ*_ for the *gfp* expression in *S*. *aureus*. Notably, the GFPmut2 was constitutively expressed in the laboratory strain RN4220^18–20^. This study therefore developed fluorescent protein expression vectors with the *P*_*blaZ*_ promoter to highly express GFP and RCFP variants in clinically isolated *S*. *aureus*.

Here, we describe novel fluorescent protein expression vectors, which were shown to exhibit greater fluorescence intensity in clinical *S*. *aureus* strains. To improve the expression of an exogenous fluorescent protein in *S*. *aureus* clinical strains, we used the *blaZ*/*sodp*(Con) promoter and codon-optimized fluorescent protein genes, and showed that the fluorescence intensity in the series of GFP and RCFP variants were significantly enhanced in the clinical strains. These new tools efficiently expressing fluorescent proteins in clinical *S*. *aureus* strains are valuable for understanding the pathogenic mechanism of *S*. *aureus*.

## Results

### Adaptation of the green fluorescent protein to *S*. *aureus*

**Figure 1:**
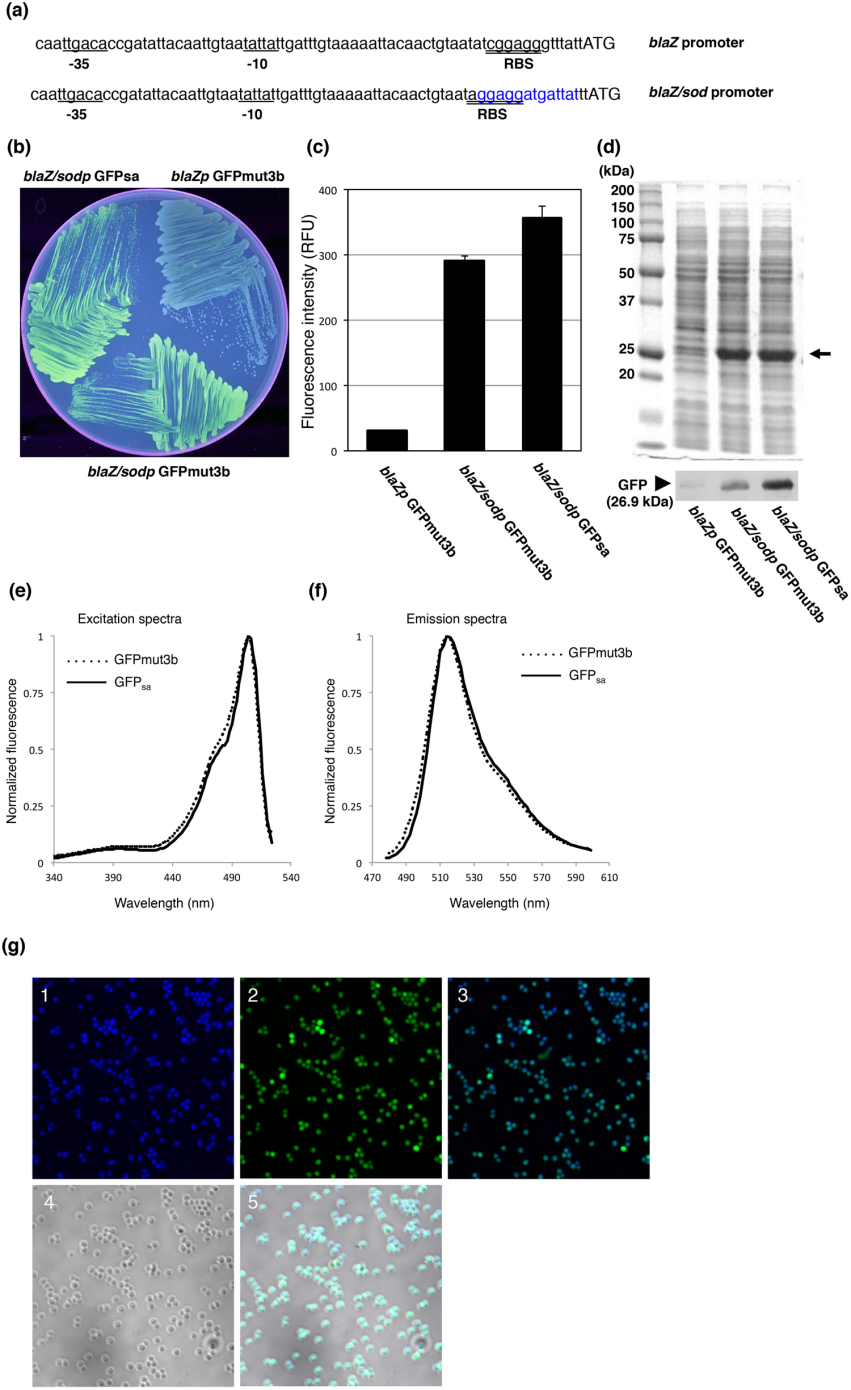
Replacement of RBS sequence and introduction of Cycle3 mutations into GFPmut3. (**a**) Sequence alignment of the *blaZp* and its derivative *blaZ*/*sodp* promoters. Blue bold face indicates the surrounding sequence containing RBS derived from the *sod* gene. Underlined sequences indicate −35 and −10 elements of the *blaZ* promoter. Double underlines indicate the RBS sequence. Initiation codons are shown in uppercase. (**b**) The fluorescing colonies were photographed under UV excitation. *S*. *aureus* RN4220 containing pS1GFP, pFK51, and pFK52 were grown on TSB agar plate containing chloramphenicol. (**c**) The fluorescent intensities of GFPmut3b, and GFP_sa_ in *S*. *aureus* RN4220. Cell homogenate of *S*. *aureus* containing pS1GFP, pFKS1 and pFK52 was prepared and the fluorescent intensities at 513 nm were measured with a microplate reader, λ_ex_ = 490 nm. The data represent mean values ± standard deviation. (**d**) The expression efficiency of pS1GFP, pFK51, and pFK52 in *S*. *aureus* RN4220. SDS-PAGE and Western blot analysis show the relative quantities of the GFP in the whole cell lysates. The Western blot gel was cropped and the full-length image is included in Supplemental Fig. 5(a). (**e**) Excitation and (**f**) Emission spectra for GFPmut3b and GFP_sa_ in *S*. *aureus* RN4220. Each spectrum was normalized to a maximum value of 1. Excitation spectra were recorded with emission at 540 nm. Emission spectra were recorded with excitation at 460 nm. GFPmut3b and GFP_sa_ were depicted by a solid line and dotted line, respectively. (**g**) *S*. *aureus* RN4220 containing pMK4*blaZ*/*sodp*GFP_sa_ (pFK52) was grown on TSB containing chloramphenicol and visualized using an FV1000 confocal scanning laser microscope (Olympus). Panels show (1) DAPI, (2) GFP, (3) overlay of DAPI and GFP images, (4) differential interference contrast (DIC), (5) overlay of DAPI, GFP, and DIC images.

We aimed to develop fluorescent protein expression vectors that can produce sufficient fluorescence intensity to visually identify fluorescing colonies of *S*. *aureus* on agar plates with the naked eye. *S*. *aureus* strain RN4220 colonies containing pS1GFP in which GFPmut3b was expressed under the control of the β-lactamase gene (*blaZ*) promoter exhibited faint fluorescence on TSB agar plates (Fig. 1b). When we replaced the 13-bp sequence containing RBS sequence of the *blaZ* gene with the corresponding region of the superoxide dismutase gene (*sod*) (Fig. 1a) that has been reported to enhance the expression of fluorescent protein in *S*. *aureus* ^13^, the fluorescent activity of the colonies expressing GFPmut3b was significantly improved on the agar plates (Fig. 1b). Further, the fluorescence intensity increased by an approximately 10-fold using the microplate assay reader (Fig. 1c). The high expression of GFPmut3b was confirmed as a major protein band corresponding to its molecular weight, 26.8 kDa, in SDS-PAGE (Fig. 1d). Although the GFPmut3b is one of the faster folding GFP variants that has been optimized for bacteria and has minimal toxicity^21^, overexpression of GFPmut3b in *S*. *aureus* showed adverse effects such as cell growth inhibition, producing smaller colonies, and frequent co-occurrence of larger colonies exhibiting no fluorescence signal (Fig. S1a). To overcome these adverse effects, GFPmut3b was modified by introducing Cycle3 mutations (F99S/M153T/V163A) that are known to improve the solubility and reduce the toxicity^9, 22^, yielding *S*. *aureus*-adapted GFPmut3b, GFP_sa_ (S65G/S72A/F99S/M153T/V163A). As a result, the mutations antagonized the appearance of non-fluorescent colonies and maintained high levels of the fluorescent intensity (Figs 1b,c and S1b) and GFP_sa_ expression (Fig. 1d) without affecting its excitation and emission wavelengths (Fig. 1e,f). Further, confocal laser scanning microscopic analysis revealed that almost all of *S*. *aureus* cells expressed GFP_sa_ protein and were clearly visualized (Fig. 1g).

### Introducing suppressor mutations into the *blaZ* gene promoter

**Figure 2:**
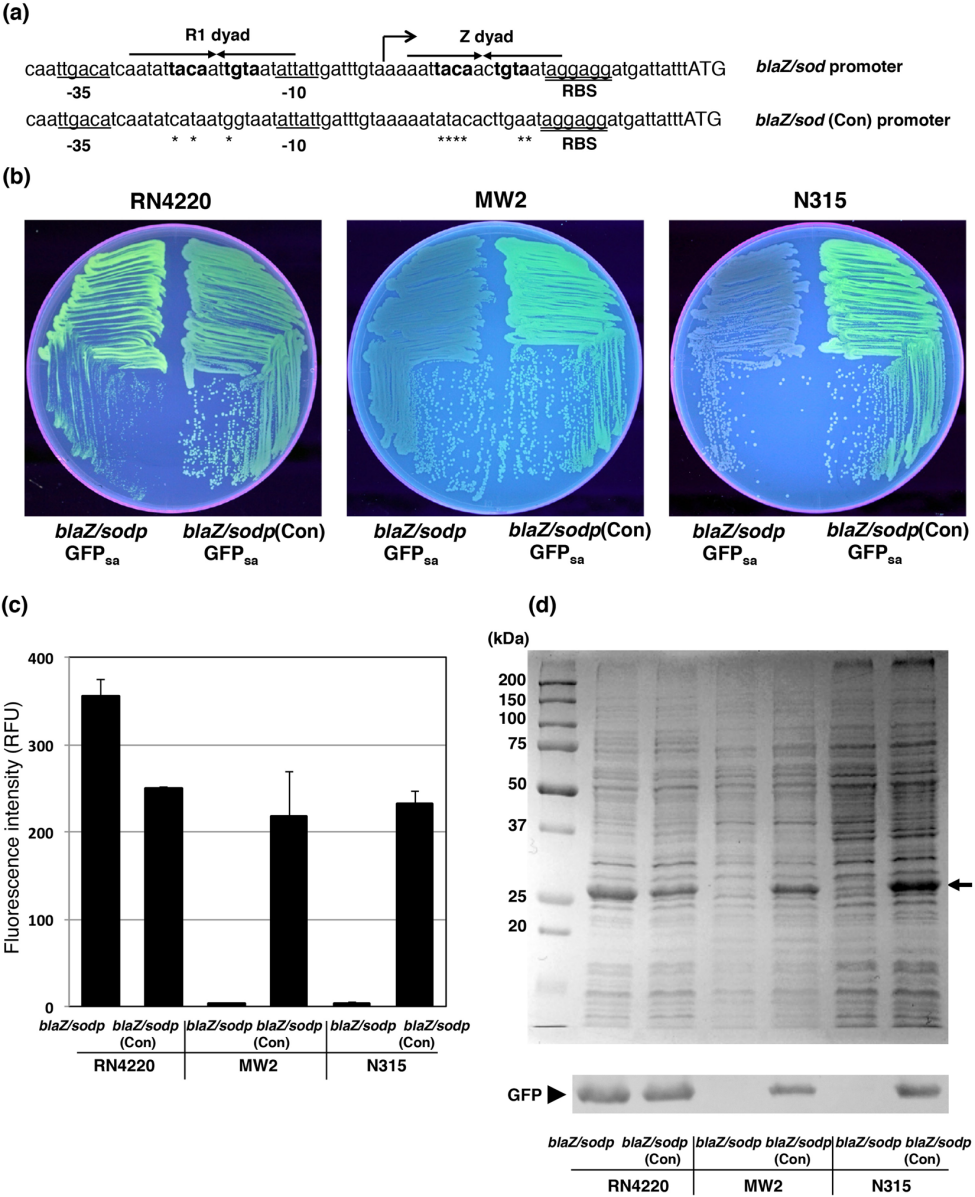
Site-directed mutagenesis into the BlaI/MecI binding sequence. (**a**) Sequence alignment of the *blaZ*/*sodp* and its constitutively induced *blaZ*/*sodp*(Con) promoter region. Asterisks indicate the sequence exchanged by site-directed mutagenesis. Bold face indicates the BlaI/MecI binding motif (TACA/TGTA) within the larger palindromes, the R1 dyad and Z dyad are indicated by arrows. Up arrows with the tip to right indicates the transcription initiation site. Underlined sequences indicate −35 and −10 elements. Double underline indicates the RBS sequence. Initiation codons are shown in uppercase. (**b**) The fluorescing colonies were photographed under UV excitation. *S*. *aureus* RN4220, MW2 and N315 containing either pFK52, or pFK54 were grown on TSB agar plate containing chloramphenicol. (**c**) The comparison of fluorescence intensity among *S*. *aureus* strain RN4220, MW2, and N315 containing either pFK52, or pFK54. The fluorescent intensities at 513 nm were measured with a microplate reader, λ_ex_ = 490 nm. The data represent mean values ± standard deviation. (**d**) The comparison of GFP_sa_ expression efficiency among *S*. *aureus* strain RN4220, MW2, and N315 containing either pFK52, or pFK54. SDS-PAGE and Western blot analysis show the relative quantities of GFP_sa_ in the whole cell lysates. The arrow indicates the position of GFP_sa_ in gel. The Western blot gel was cropped and the full-length image is included in Supplemental Fig. 5(b).

*S*. *aureus*-adapted GFP_sa_ demonstrated higher expression under the control of the hybrid *blaZ*/*sodp* promoter in laboratory strain RN4220. However, in clinical strains, MW2 and N315, with the same vector, the expression of GFP_sa_ was very low, and the fluorescence intensity was poor (Fig. 2b,c,d). BlaZ plasmid is prevalent in clinical strains^23, 24^, and the *blaIRZ* gene operon is natively present on a plasmid in both clinical strains, MW2 and N315, but not in RN4220^25–27^. The BlaI and MecI specifically bind to the same dyad symmetry (TACA/TGTA) sequence and repress the *blaZ* promoter activity (Fig. 2a)^28^. To minimize the possible negative effect by the endogenous BlaI/MecI on the *blaZ*/*sodp* promoter activity, we introduced suppressor mutations into the BlaI/MecI binding motif (TACA/TGTA), yielding a constitutively induced *blaZ*/*sodp*(Con) promoter (Fig. 2a). As a consequence, GFP_sa_ expression was significantly elevated, and the resulting fluorescence intensity was highly improved in both N315 and MW2, whereas the fluorescence intensity was slightly reduced in strain RN4220, compared with the parental sequence (Fig. 2b,c,d). Taken together, the data demonstrated the modified *blaZ*/*sodp* promoter, (*blaZ*/*sodp*(Con)) can effectively enhance the expression of GFP_sa_ in *S*. *aureus* clinical strains.

### Expression of multicolor GFP variants in clinical strains

**Figure 3:**
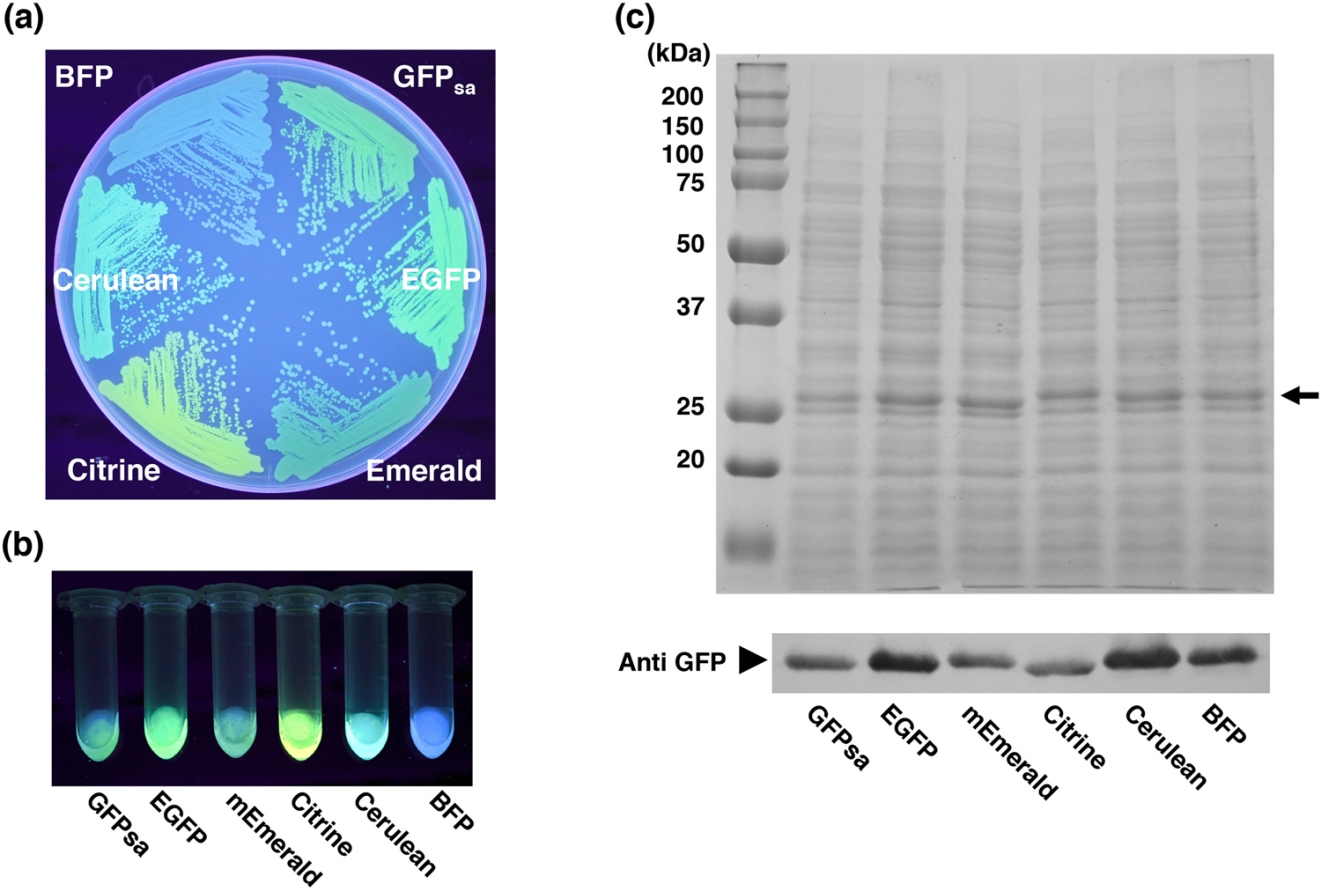
Detection of multicolor GFP variants in the clinical strain, MW2. (**a**) The fluorescing colonies were photographed using UV excitation. *S*. *aureus* strain MW2 expressing multicolor GFP variants (GFP_sa_, EGFP, mEmerald, Citrine, Cerulean, and BFP) were grown on TSB agar plate containing chloramphenicol. (**b**) The whole cell lysates of *S*. *aureus* MW2 expressing multicolor variants used for SDS-PAGE and Western blot analysis were photographed under UV excitation. (**c**) The comparison of expression efficiency among multicolor GFP variants in *S*. *aureus* strain MW2. SDS-PAGE and Western blot analysis show the relative quantities of the multicolor GFP variants in the whole cell lysates. The arrow indicates the position of GFP variants in gel. The Western blot gel was cropped and the full-length image is included in Supplemental Fig. 5(c).

*A*. *victoria* GFP and its variants contribute to multicolor imaging with spectral profiles ranging in color from blue to yellow, and have been engineered by site-directed mutagenesis^29–34^. Using GFP_sa_ as the standard, we next constructed these GFP color variants (EGFP, mEmerald, Citrine, Cerulean, and BFP) using amino acid substitutions considering the codon usage patterns in *S*. *aureus*. Since several BFP variants were engineered using substitution of the T66H mutation; we constructed three BFP variants, EBFP (F64L/S65T/Y66H/Y145F), 1EMF (F64L/Y66H/V163A), and P4–3E (F64L/Y66H/Y145F/V163A) and evaluated which BFP variants would be more suitable for *S*. *aureus* MW2 and N315. Fluorescence assay showed that homogenates of P4-3E and EBFP exhibited somewhat greater fluorescence intensity than 1EMF when *S*. *aureus* MW2 were grown in TSB medium (Fig. S2a), while only *S*. *aureus* expressing P4-3E was visually identified as fluorescing colonies on agar plates by the naked eye (Fig. S2b). Each strain expressing the multicolor GFP variant exhibited sufficient fluorescence intensity to visually identify fluorescing colonies by the naked eye (Fig. 3a), and SDS-PAGE and Western blot analyses showed that like GFP_sa_ all of these multicolor variants were expressed with high yield in *S*. *aureus* MW2 harboring the corresponding vectors with the *blaZ*/*sodp*(Con) promoter (Fig. 3b,c). The multicolor GFP variant genes driven by the *blaZ*/*sodp*(Con) promoter also exhibited high fluorescence intensity in another clinical strain, N315 (Fig. S3a). Further, fluorescence excitation and emission spectrum analysis showed these strains expressing the multicolor GFP variants reflected the previously published spectrum profiles (Fig. S4). This data indicated that multicolor GFP variants were highly expressed in clinical strains MW2 and N315 with the *blaZ*/*sodp*(Con) promoter.

### Codon optimization of AmCyan and mCherry for *S*. *aureus*

**Figure 4:**
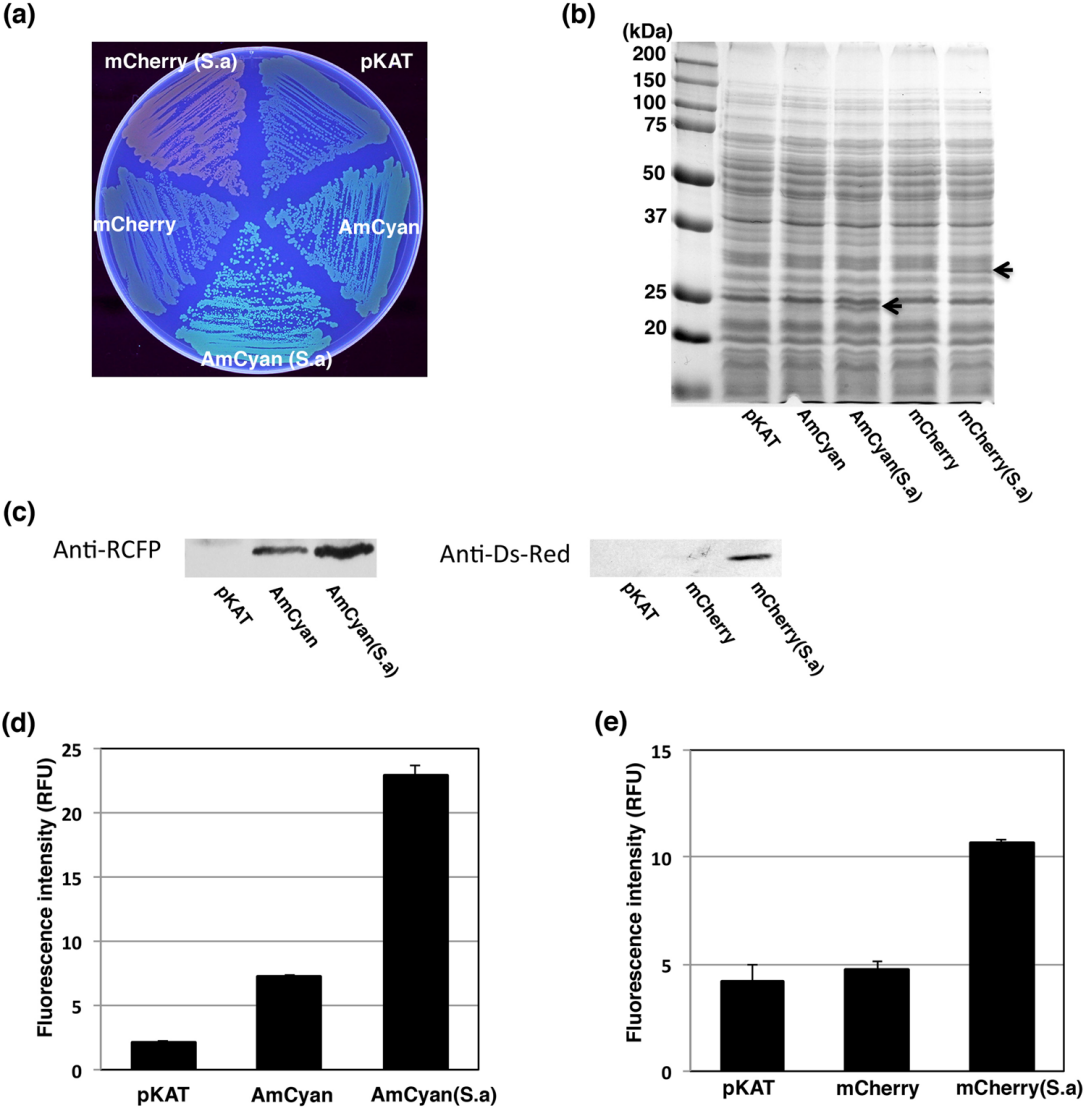
Codon usage optimization of the *amCyan* and *mCherry* genes. (**a**) The fluorescing colonies were photographed under UV excitation. *S*. *aureus* strain MW2 containing pKAT (control), pFK62 (AmCyan), pFK64 (AmCyan(S.a)), pFK63 (mCherry), and pFK65 (mCherry(S.a)) were grown on TSB agar plates containing chloramphenicol. (**b**) SDS-PAGE analysis showed the relative quantities of AmCyan and mCherry in the whole cell lysates. Arrows indicate the position of AmCyan and mCherry in gel. (**c**) Western blot analysis of AmCyan or mCherry in the whole cell lysates. The Western blot gels were cropped and the full-length image are included in Supplemental Fig. 5(d). The comparison of fluorescent intensities of pKAT (control), pFK62 (AmCyan), and pFK64 (AmCyan(S.a)) in *S*. *aureus* MW2. The fluorescent intensities at 489 nm were measured with a microplate reader, λ_ex_ = 458 nm. The data represent mean values ± standard deviation. (**e**) The comparison of fluorescent intensities of pKAT (control), pFK63 (mCherry), and pFK65 (mChaerry(S.a)) in *S*. *aureus* MW2. The fluorescent intensities at 610 nm were measured with a microplate reader, λ_ex_ = 586 nm. The data represent mean values ± standard deviation.

Like *Aequorea* GFP, reef coral fluorescent proteins (RCFP) and their variants also contribute to multicolor imaging with spectral profiles ranging in color from cyan to far red^1, 5^. We constructed the RCFP expression vectors for *S*. *aureus* clinical strains, in which the *amCyan* or *mCherry* gene was expressed under the control of the hybrid *blaZ*/*sodp*(Con) promoter. Unexpectedly, *S*. *aureus* expressing the commercially available *amCyan* or *mCherry* gene formed faintly fluorescent colonies on agar plates (Fig. 4a), we therefore optimized the codon usage of *amCyan* and *mCherry* genes by replacing rare GC- rich codons with AT-rich ones. As a consequence, the codon-optimized AmCyan (AmCyan(S.a)) and mCherry (mCherry(S.a)) exhibited sufficient fluorescence intensity to visually identify fluorescing colonies on agar plates by the naked eye (Fig. 4a), and the corresponding proteins were confirmed to their molecular weights, 25.2 kDa and 26.7 kDa, respectively (Fig. 4b) and detected by Western blot (Fig. 4c). The fluorescence assay shows the codon adapted genes resulted in increased fluorescence intensity of AmCyan and mCherry (Fig. 4d,e). The *amCyan* and *mCherry* genes driven by the *blaZ*/*sodp*(Con) promoter also exhibited a significant fluorescence intensity in strain N315 (Fig. S3b). Further, fluorescence excitation and emission spectrum analysis showed these strains expressing AmCyan or mCherry reflected previously published spectrum profiles (Fig. S4). Taken together, the expression of AmCyan and mCherry were significantly improved in *S*. *aureus* clinical strains.

### Identification of *S*. *aureus* cells expressing a specific fluorescent protein in co-culture systems

**Figure 5:**
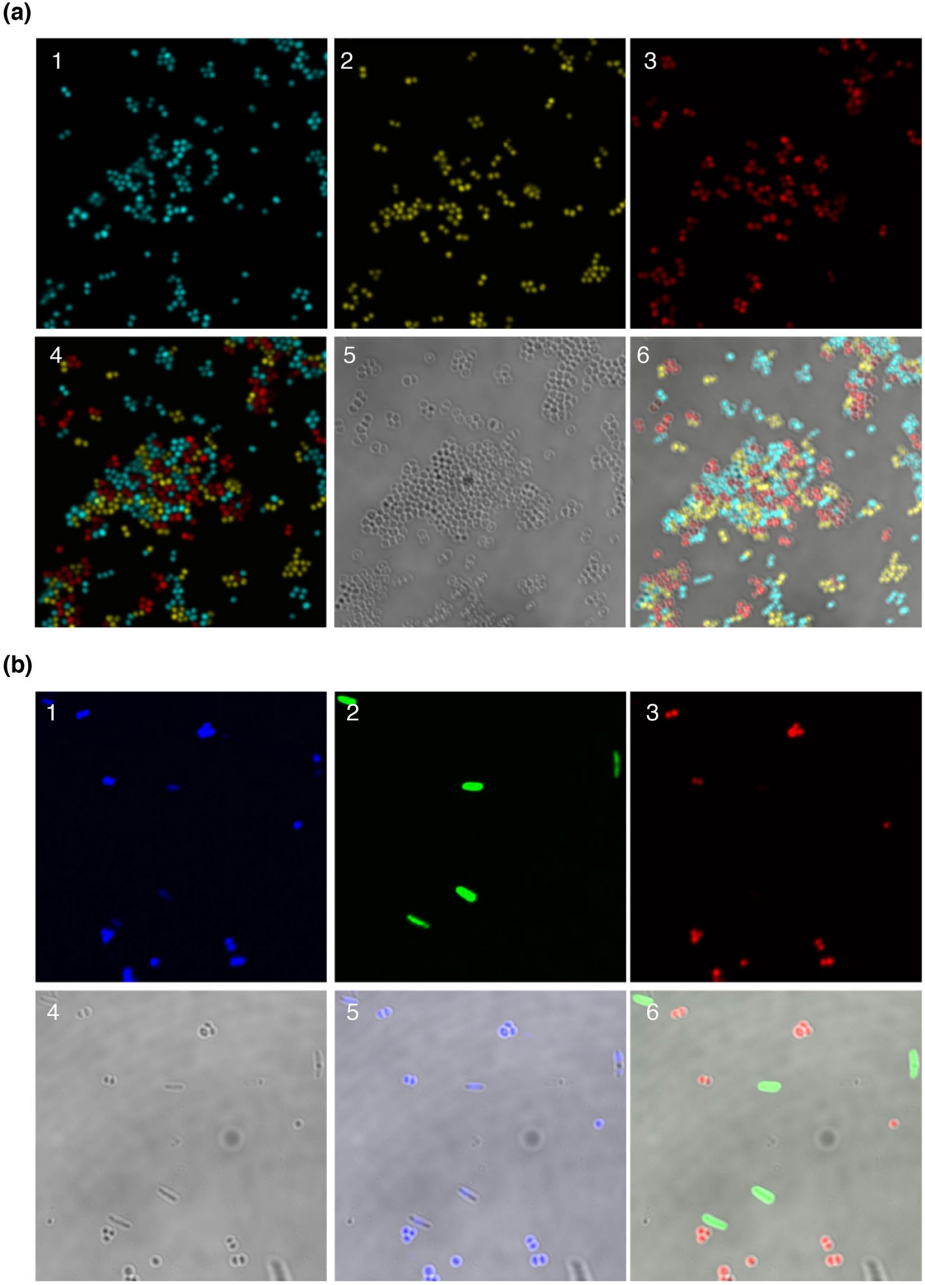
Confocal laser microscopic analysis in co-culture systems. (**a**) Co-culture of three clinically isolated *S*. *aureus* strains. *S*. *aureus* strain N315 containing pFK60 (Cerulean), strain TY34 containing pFK56 (Citrine), and strain MW2 containing pFK65 (mCherry(S.a)) were co-cultured in BHI broth. Panels show (1) Cerulean (N315), (2) Citrine (TY34), (3) mCherry(S.a) (MW2), (4) overlay of Cerulean, Citrine, and mCherry images, (5) DIC, (6) overlay of Cerulean, Citrine, mCherry, and DIC images. (**b**) Co-culture of *S*. *aureus* with *E*. *coli*. *S*. *aureus* N315 containing pFK65 (mCherry(S.a)) and *E*. *coli* DH5α containing pFK55 (EGFP) were co-cultured in BHI broth. Panels show (1) DAPI, (2) EGFP, (3) mCherry(S.a), (4) DIC, (5) overlay of DAPI and DIC images, and (6) overlay of EGFP, mCherry(S.a), and DIC images.

We next aimed to discriminate *S*. *aureus* cells expressing a specific fluorescent protein from the co-existence of bacteria expressing different fluorescent proteins in a co-culture experiment. Confocal laser scanning microscopic analysis revealed that each *S*. *aureus* clinical strain expressing different fluorescent proteins was selectively detected in the co-culture system in which Cerulean, Citrine, and mCherry(S.a) were differently expressed in the strain N315, TY34^35^, and MW2, respectively (Fig. 5a). Further, *S*. *aureus* N315 cells expressing mCheery(S.a) were clearly distinguished from *E*. *coli* DH5α cells expressing EGFP (Fig. 5b). These results demonstrated that our fluorescent protein expression vectors have the potential to sensitively detect particular *S*. *aureus* cells in co-culture with different strains or other bacteria.

## Discussion

In this study, we developed new fluorescent protein vectors to express a bright fluorescence intensity enough to visually identify fluorescent colonies of *S*. *aureus* macroscopically on agar plates. Practically available high-level expression of fluorescent protein has not been achieved in previous studies in which fluorescent proteins were detected only with fluorescence microscopy^10–14^. Our findings provide new applications to enhance the expression of multicolor GFP variants and would allow us to sensitively detect particular *S*. *aureus* cells in bacterial populations and in animal infection models.

To enhance fluorescent brightness, we replaced the sequence from the Shine-Dalgarno (SD) sequence to the start codon with *sod* that was previously reported with the *sod* RBS to lead to the highest fluorescence intensity with the *sarA* promoter^13^. Consequentially, we not only modified the SD sequence of *blaZ* gene to an optimal SD sequence (AGGAGG), but also altered the distance from the SD sequence to the start codon. The distance between the RBS and the start codon is also known to affect the efficiency of translation initiation^36^. Our results support the concept that the improvement of the sequence from the SD sequence to the start codon sequence significantly enhances the expression of the GFP variant, although the exact mechanism responsible for this effect remains unclear (Fig. 1b). However, recent studies suggest the interaction among the RBS, the initiation codon, the 5′-coding region in translation initiation, and RNA secondary structure at the 5′ terminus affects the protein expression in bacteria^37, 38^. Therefore, the enhancement of GFP production may be accounted for by the decrease of free fold energy of the 5′ end of mRNA transcripts.

At the beginning of this study, we evaluated GFP_sa_ expression vectors with the *blaZ*/*sodp* promoter in a laboratory strain, RN4220. This strain is easily genetically manipulated; however, it carries a number of genetic mutations that may affect the virulence of the strain^20, 27^. Like RN4220, laboratory strains may not be suitable for the evaluation of pathogenesis, because laboratory strains often lack important pathophysiological characters^39^. Hence, clinical strains should be used to properly evaluate the pathogenesis and virulence of *S*. *aureus*. *S*. *aureus* harboring the *bla* gene locus appeared in the 1940s after the introduction of penicillin; and at present, most clinical isolates carry the *blaIRZ* gene on plasmids or on the chromosome^23, 40, 41^. Because the GFP_sa_ expression driven by the *blaZ*/*sodp* promoter was not detectable in *S*. *aureus* clinical strains, MW2 and N315 (Fig. 2), we hypothesized the endogenous BlaI may inhibit the transcription of the *blaZ*/*sodp* promoter in trans; and we therefore generated the constitutively induced *blaZ*/*sodp*(Con) promoter to overcome the limitation of the clinical strains. The *blaZ*/*sodp*(Con) promoter can be adapted to enable expression of not only fluorescent proteins but also various exogenous proteins (toxins) from clinical *S*. *aureus* strains.

Codon optimization frequently plays a key role in exogenous protein expression^37, 42^. The GC contents of human codon-optimized *amCyan* and *mCherry* are 46.8% and 62.5%, respectively, showing strong preferences for G + C at the third codon position is distinct from the codon usage patterns in *S*. *aureus* with 33.5% GC content. Exchanging the 92 nucleotides of *amCyan* gene and 76 nucleotides of *mCherry* gene, respectively, both fluorescence intensities were significantly improved overcoming the codon usage bias in *S*. *aureus*. However, SDS-PAGE analysis showed the level of RCFPs production did not reach the level of GFP variants (Figs 3c and 4b). These results suggest the possibility that further investigation with these vectors could improve the expression level of RCFPs by further adapting of codon usage or decreasing the free folding energy of the initial 5′-coding region.

In summary, we have developed new multicolor fluorescent protein vectors that efficiently enhance fluorescence intensity in *S*. *aureus* clinical strains where the greater fluorescence intensity of multicolor fluorescent proteins may facilitate clear visualization of *S*. *aureus* clinical strains. Ultimately, these findings may help in better understanding the pathogenic mechanisms of *S*. *aureus*.

## Methods

### Bacterial strains, plasmids and growth conditions

The bacterial strains and plasmids used in this study are in Table 1. *S*. *aureus* and *E*. *coli* were cultured at 37 °C with shaking at 140 rpm in test tube (25 mm × 150 mm) containing 3 ml of trypticase soy broth (TSB) (Becton, Dickinson and Company) or 3 ml of lysogeny broth (5 g yeast extract, 10 g polypeptone, 10 g NaCl per liter; pH 7.2), respectively. Ampicillin (Amp, 100 µg/ml) and chloramphenicol (Cp, 10 µg/ml) were added to the medium if necessary. The plasmids encoding AmCyan or mCherry were purchased from Clontech, TaKaRa Bio Inc., Japan.

**Table 1:** Bacterial strains and plasmids used in this study.

Bacterial strain or plasmid	Relevant characteristic(s)	Source or reference
*E. coli*		
DH5*α*	F^−^, φ80d*lacZ*ΔM15, Δ(*lacZYA*−*argF*)U169, *deoR*, *recA*1, *endA*1, *hsdR*17(rk^−^, mk^+^), *phoA*, *supE*44, λ^−^, *thi*-1, *gyrA*96, *relA*1	TaKaRa
*S. aureus*		
RN4220	NCTC8325-4, r^-^ m^+^	20
N315	hospital-aquired MRSA	25
MW2	community-aquired MRSA	26
TY34	clinical isolate MRSA from patient with impetigo	35
Plasmids		
pMK4	Shuttle vector between *E. coli* and *S. aureus*, Am^r^ in *E. coli*, Cm^r^ in *S. aureus*	46
pS1GFP	pMK4 containing GFPmut3b gene fused to the *blaZp* promoter	19
pND50	Shuttle vector between *E. coli* and *S. aureus*, Cm^r^	44
pKAT	pND50 derivative containing the *lacZ(α)* gene from pUC19, Cm^r^	43
pAmCyan	Plasmid encoding AmCyan gene, Amp^r^	TaKaRa Clontech
pmCherry	Plasmid encoding mCherry gene, Amp^r^	TaKaRa Clontech
pFK51	pMK4 containing GFPmut3b gene fused to the *blaZ/sodp* promoter	This study
pFK52	pMK4 containing GFPsa gene fused to the *blaZ/sodp* promoter	This study
pFK53	pMK4 containing GFPmut3b gene fused to the *blaZ/sodp*(Con) promoter	This study
pFK54	pMK4 containing GFPsa gene fused to the *blaZ/sodp*(Con) promoter	This study
pFK55	pMK4 containing EGFP gene fused to the *blaZ/sodp*(Con) promoter	This study
pFK56	pMK4 containing Citrine gene fused to the *blaZ/sodp*(Con) promoter	This study
pFK57	pMK4 containing EBFP gene fused to the *blaZ/sodp*(Con) promoter	This study
pFK58	pMK4 containing BFP(P4-3E) gene fused to the *blaZ/sodp*(Con) promoter	This study
pFK59	pMK4 containing BFP(1EMF) gene fused to the *blaZ/sodp*(Con) promoter	This study
pFK60	pMK4 containing Cerulean gene fused to the *blaZ/sodp*(Con) promoter	This study
pFK61	pMK4 containing mEmerald gene fused to the *blaZ/sodp*(Con) promoter	This study
pFK62	pKAT containing AmCyan gene fused to the *blaZ/sodp*(Con) promoter	This study
pFK63	pKAT containing mCherry gene fused to the *blaZ/sodp*(Con) promoter	This study
pFK64	pKAT containing codon-optimized AmCyan(S.a) gene fused to the *blaZ/sodp*(Con) promoter	This study
pFK65	pKAT containing codon-optimized mCherry(S.a) gene fused to the *blaZ/sodp*(Con) promoter	This study

### Improvement of GFP expression vector using site-directed mutagenesis

To increase expression of fluorescent proteins in *S*. *aureus* clinical strains, we improved a GFP expression vector based on pS1GFP carrying GFPmut3b gene (a kind gift from Prof. M. Krönke)^19^. The hybrid *blaZ*/*sodp* promoter was constructed by replacing the sequence containing the RBS in the *blaZ* gene in the *sod* gene with blaZPR and GFP-F primers using the inverse PCR method with KOD Plus Neo DNA polymerase (TOYOBO, Japan). The PCR product was phosphorylated with T4 Polynucleotide Kinase (TaKaRa Bio Inc, Japan), and circularized by self-ligation with Ligation high Ver.2 (TOYOBO, Japan); and then the circular DNA was transformed into *E*. *coli* DH5α. Plasmid DNA was extracted from the transformed *E*. *coli* DH5α using FastGene^TM^ Plasmid Mini Kit (Nippon Genetics Co., Ltd. Japan) and the resultant plasmid was verified using ABI 3130 DNA sequencer (Applied Biosystems). To express the fluorescent protein in *S*. *aureus* clinical strains, the substitution of the repressor BlaI/MecI binding motif (TACA/TGTA) was performed with the blaZ-mutF and blaZ-mutR primers using inverse PCR as above, yielding the constitutively induced *blaZ*/*sodp*(Con) promoter.

### Construction of the multicolor GFP variants using amino acid substitution

Amino acid substitution was performed using inverse PCR with pMK4*blaZ*/*sodp*(Con)GFPmut3b (pFK53) as the initial template with the KOD Plus Neo DNA polymerase (TOYOBO, Japan). The primers described in Table 2 were used to construct the GFP variants (Table 3). The resulting plasmids were confirmed using DNA sequencing.

**Table 2:** Oligonucleotide primers.

Name	primer sequence (5' to 3')	Purpose
blaZPR	AAATAATCATCCTCCTATTACAGTTGTAA	replacement of RBS sequence
GFP-F	ATGAGTAAAGGAGAAGAACTTTTCAC	
blaZPmut-F	TTTGTAAAAATATACACTTGAATAGGAGGATGAT	destruction of BlaI motif
blaZPmut-R	TCAATAATATTACCATTATGATATTGATG	
F64LS65T-R	TTGAACACCATATGTTAAAGTAGTAACAAG	construction of Emerald and EGFP
F64LY66H-R	TTGAACACCATGAGATAAAGTAGTAACAAG	construction of BFP(P4-3E) and BFP(1EMF)
F64LS65TY66H-R	TTGAACACCATGTGTTAATGTAGTAACAAG	construction of EBFP
F64LS65TY66W-R	TTGAACACCCCATGTTAAAGTAGTAACAAGTGTTG	construction of Cerulean
S65GV68LQ69M-R	CATTAAACCATAACCAAATGTAGTAACAAG	construction of Citrine
S72-F	TGTTTTTCAAGATATCCAGATCATATG	construction of EGFP and BFP
S72A-F	TGTTTTGCAAGATATCCAGATCATATG	construction of Cerulean, Emerald, and Citrine
F99S-F	TTCAAAGATGACGGTAACTACAAGAC	construction of GFPsa
F99S-R	AGATATAGTTCTTTCCTGTACATAACC	construction of GFPsa
Y145F-R	GATATATACATTATGTGAGTTAAAGTTATATTC	construction of EBFP, P4-3E(BFP)
Y145AN146IH148D-R	AATATATACATTGTCTGAGATAGCGTTATATTCCAA	construction of Cerulean
N149KM153T-R	CTTTTGTTTGTCTGCTGTAATATATACTTTATGTGAG	construction of Emerald
I152-R	AATATATACATTATGTGAATTATAGTTATATTCC	construction of GFPsa and BFP(1EMF)
M153-F	ATGGCAGACAAACAAAAGAATGGAATC	construction of EBFP, P4-3E(BFP)
M153TV163A-F	ACAGCAGACAAACAAAAGAATGGAATCAAAGCTAAC	construction of GFPsa and Cerulean
V163A-F	ATGGCAGACAAACAAAAGAATGGAATCAAAGCTAAC	construction of 1EMF(BFP)
I167T-F	AATGGAATTAAAGTTAACTTCAAAACAAGACAC	construction of Emerald
T203Y-F	TATCAATCTGCATTATCAAAAGATCCAAAC	construction of Citrine
T203Y-R	TGACAAATAATGATTGTCTGGTAAAAGAAC	construction of Citrine
A206K-F	ACACAATCTAAATTATCAAAAGATCCAAACG	monomerization
gapRF	AGAGAGGATCCTTAAATAGTTAGTTG	amplification of gapR promoter
gapRGFPR	GAAAAGTTCTTCTCCTTTACTCATTACTACCTCCTCCTTATATTTATA	amplification of gapR promoter fused to GFP
gapRGFPF	TATAAATATAAGGAGGAGGTAGTAATGAGTAAAGGAGAAGAACTTTTC	amplification of gfp gene fused to gapR promoter
GFPRB	GTCTAGATCTTTATTTGTATAGTTCATC	amplification of gfp gene
blaZPF	ACAAAAGCTTACTATGCTCATTATTAA	amplification of blaZ gene promoter
blaZPR-Cyan	AACTTGTTTGAAAGAGCCATAAATAATCATCCTCCTATTA	amplification of blaZ gene promoter fused to AmCyan
blaZP-CyanF	TAATAGGAGGATGATTATTTATGGCTCTTTCAAACAAGTT	amplification of amCyan gene fused to blaZ promoter
blaZPR-mCherry	TCCTCGCCCTTGCTCACCATAAATAATCATCCTTCCTATTA	amplification of blaZ gene promoter fused to mCherry
blaZP-mCherryF	TAATAGGAGGATGATTATTTATGGTGAGCAAGGGCGAGGA	amplification of mCherry gene fused to blaZ promoter
pUC-RH	AATGGAAGCTTCCGGCGCTCAGTTGG	amplification of amCyan and mCherry
Cyan1F	CATATGAAGGTACACAAACATCAACTTTTAAAGTTACAATGGCAAACGGTGGTCCACTTGCATTCTCATT	codon optimization for amCyan
Cyan1R	GTTTACCACTACCTTCACCTTTAACTGTAAAATAATGACCGTTAACACAACCATCCATATGATATGT	
Cyan2F	ATGCCAGATTATTTTAAACAAGCATTTCCTGATGGTATGTCATATGAACGTACTTTTACA	codon optimization for amCyan
Cyan2R	ACTTGTAGGATATGCAGTAAAACAACGATTACCATACATAAAAACTGTTGATAGAATAC	
Cyan3F	GAACATAAATCAACATTTCATGGAGTTAACTTTCC	codon optimization for amCyan
Cyan3R	AAAACAGTTACCTTTAAGACTTATTTCCCAACTTGC	
Cyan4F	CAAGGAGGTGGTAATTATAGATGTCAATTTCATACTTCTTATAAGACA	codon optimization for amCyan
Cyan4R	TAACATTAAAAATGCTGTAACATCACCCTTCAATATTCCATCACAAACAGTC	
Cyan5F	AAGGTGGTAATAGTGTTCAATTAACAGAACATGCTGTTGCACATATAACATCTGTTGTTCC	codon optimization for amCyan
Cyan5R	TATCTAAATCTGTTCTTGCAATACGATGTTCAACTGCATGGTTTGGTGGCATTGTAACTGGTTT	
Cherry1F	AGTTAATGGTCATGAATTCGAAATCGAGGGCGAGGGCGAGGGTCGTCCATATGAGGGCACACAAACAGC	codon optimization for mCherry
Cherry1R	GAACCTTCCATATGAACTTTAAAACGCATGAACTCCTTGATGATTGCCATGTTATCCTCCTCACCCTTAC	
Cherry2F	TATGGTTCAAAAGCATATGTTAAGCATCCAGCAGACATCCCAGACTATTTGAAGTTGTCATTCCCAGAGG	codon optimization for mCherry
Cherry2R	CATAAATTGAGGTGATAAGATGTCCCATGCGAATGGCAATGGACCACCCTTTGTCACCTTCAACTTTGC	
Cherry3F	TATTTATAAAGTTAAGTTGCGTGGTACAAACTTCCCATCAGACGGCCCAGTAATGCAGAAGAAGACAATG	codon optimization for mCherry
Cherry3R	AATTCACCATCTTGCAATGATGAGTCTTGTGTCACTGTAACCACACCGCCGTCCTCGAAGTTCATCACAC

**Table 3:** GFP variants used in this study.

GFP variant	Mutations relative to wtGFP	Reference
GFPmut3b	S65G, S72A	21
GFPsa	S65G, S72A, F99S, M153T, V163A	This study
EGFP	F64L, S65T	4
mEmerald	F64L, S65T, S72A, N149K, M153T, I167T, A206K	4
Citrine	S65G, S72A, V68L, Q69M, T203Y	30
Cerulean	F64L, S65T, Y66W, S72A, Y145A, N146I, H148D, M153T, V163A	31
1EMF(BFP)	F64L, Y66H, V163A	32
EBFP	F64L, S65T, Y66H, Y145F	33
P4-3E(BFP)	F64L, Y66H, Y145F, V163A	34

### Construction of codon-optimized RCFP expression vectors

The *amCyan* and *mCherry* genes were combined with the hybrid *blaZ*/*sodp*(Con) promoter using overlap extension PCR and cloned into the pKAT vector^43^. Plasmid pKAT derived from pND50^44^ is an *E*. *coli*-*S*. *aureus* shuttle vector containing the replication origins of pUB110 (*S*. *aureus*) and the pUC19 (*E*. *coli*) *lacZ*(α) gene from pUC19. This enables a simple blue-white screening for clones in *E*. *coli*, and enables the *cat* gene conferring resistance to chloramphenicol in both *E*. *coli* and *S*. *aureus*. All restriction enzyme sites in the multiple cloning site (MCS) located in the *lacZ*(α) gene can be used for cloning into pKAT. In the first PCR, the hybrid *blaZ*/*sodp*(Con) promoter region, *amCyan* gene, and *mCherry* gene were amplified from pFK53, pAmCyan (Clontech, TaKaRa Bio Inc., Japan), and pmCherry (Clontech, TaKaRa Bio Inc., Japan) with the following primer sets (AmCyan: blaZp-F and blaZPR-Cyan and blaZP-CyanF and pUC-RH; mCherry: blaZp-F and blaZPR-Cherry and blaZP-mCherryF and pUC-RH), respectively. The second PCR was performed with the mixture of two PCR fragments as the template using the primer set (blaZp-F and pUC-RH). The resulting PCR products were digested with HindIII and cloned into the same site in pKAT. Codon optimization was then repeatedly performed using inverse PCR as mentioned above with the primers described in Table 2 that were designed to optimize the codon usage of *amCyan* and *mCherry* gene using the Kazusa Codon Usage Database (http://www.kazusa.or.jp/codon/). The DNA sequences of codon-optimized *amCyan* and *mCherry* genes have been submitted to the GenBank and are available under accession numbers LC088723 (amCyan) and LC88724 (mCherry).

### Transformation of *S*. *aureus* and quantification of fluorescence intensity

In brief, *S*. *aureus* was individually transformed using electroporation as described previously^45^. Each plasmid was first transformed into *S*. *aureus* RN4220^20^ and selected as chloramphenicol-resistant colonies, then the resulting modified plasmids were isolated and electroporated into *S*. *aureus* MW2 and N315. *S*. *aureus* was grown as described in the growth conditions. Bacterial cells from overnight cultures were washed with phosphate-buffered saline (PBS) and re-suspended to an optical density at 660 nm of 0.2. The cell suspension was then dispensed into triplicate wells (100 µl/well) of a U-bottom 96 well cell culture plate (Greiner Bio-One). The fluorescence intensity was measured using a Varioskan Flash Multimode Reader (ThermoFisher Scientific Inc.) with two independent samples.

### Quantification of fluorescent proteins using SDS-PAGE and Western blot analysis

Fluorescent proteins were detected from whole cell lysates. *S*. *aureus* was cultured overnight as described in the growth conditions. The pre-cultured cells were adjusted to an optical density at 660 nm of 0.02 with TSB with chloramphenicol, and 3 ml of the culture were transferred into test tube (25 mm × 150 mm) and then incubated at 37 °C with shaking at 140 rpm for 16 h. The bacterial cells from 1 ml cultures were harvested by centrifugation, and then the whole cell lysates were prepared as follows: cells were re-suspended in 200 μl CS buffer (100 mM Tris-HCI, 150 mM NaCl, 100 mM EDTA, pH7.5) containing 1 μg of lysostaphin (Wako Pure Chemical Industries, Co., Ltd, Japan) and incubated at 37 °C for 30 min. Ten microliters of cell lysates were separated on SDS–PAGE and stained with Coomassie Brilliant Blue (CBB), and the fluorescent proteins were detected using Western blot with the following antibodies: Anti-GFP-HRP-Direct T (MBL Co., Ltd.), Living colors® Anti-RCFP Polyclonal Pan Antibody (Clontech Laboratories) and DsRed Polyclonal Antibody (Clontech Laboratories) as the primary antibody, and HRP-conjugated goat antibodies against rabbit IgG (MP Biomedicals, LLC-Cappel Products) as the secondary antibody. Immuno-detection of protein was performed on Pierce® Western Blot Substrate (Thermo Fisher Scientific Inc.) with X-ray film.

### Confocal laser scanning microscopic analysis

*S*. *aureus* strains and *E*. *coli* were cultured in test tube (25 mm × 150 mm) containing 3 ml of Brain Heart Infusion (BHI) broth with chloramphenicol or ampicillin at 37 °C with shaking at 140 rpm overnight. The pre-cultured cells were mixed and diluted 1:000 into 3 ml fresh BHI, and then incubated at 37 °C with shaking at 140 rpm for 6 h. Bacterial cells were harvested by centrifugation, washed twice with PBS buffer, fixed with 3% parafomaldehyde in PBS, and washed twice with PBS. Bacterial cells were counterstained with 0.1 μg/ml of 4′,6′-diamidino-2-phenylindole (DAPI) if necessary. The cell suspensions were spotted onto glass slides and air-dried at room temperature. The coverslips were mounted using VECTASHIELD H-1000 (Vector Laboratories, Inc. Burlingame, CA). All confocal images were recorded using an confocal laser scanning microscopy (Olympus, Fluoview FV1000, Japan).

## Electronic supplementary material

Supplementary Information
