# Supplementary material for: The development of fluorescent protein tracing vectors for multicolor imaging of clinically isolated Staphylococcus aureus
Source: Sci Rep. 2017 Jun 6;7:2865. doi: 10.1038/s41598-017-02930-7 (PMC5460165; doi:10.1038/s41598-017-02930-7)
Supplement: Supplementary file 1 — Supplementary Information [file 41598_2017_2930_MOESM1_ESM.pdf]

The development of fluorescent protein tracing vectors for multicolor imaging of clinically isolated *Staphylococcus aureus*.

Fuminori Kato, Motoki Nakamura, Motoyuki Sugai

Department of Bacteriology, Hiroshima University Graduate School of Biomedical and Health Sciences. Hiroshima 734-8551, Japan

*Address correspondence to: Motoyuki Sugai, Department of Bacteriology Hiroshima University Graduate School of Biomedical Sciences, Kasumi 1-2-3, Minami-ku, Hiroshima City, Hiroshima 734-8551, Japan

Tel:+81 82 257 5635 Fax:+81 82 257 5639

E-mail: sugai@hiroshima-u.ac.jp/

Supplementary Information

(a) GFPmut3b (b) GFPsa

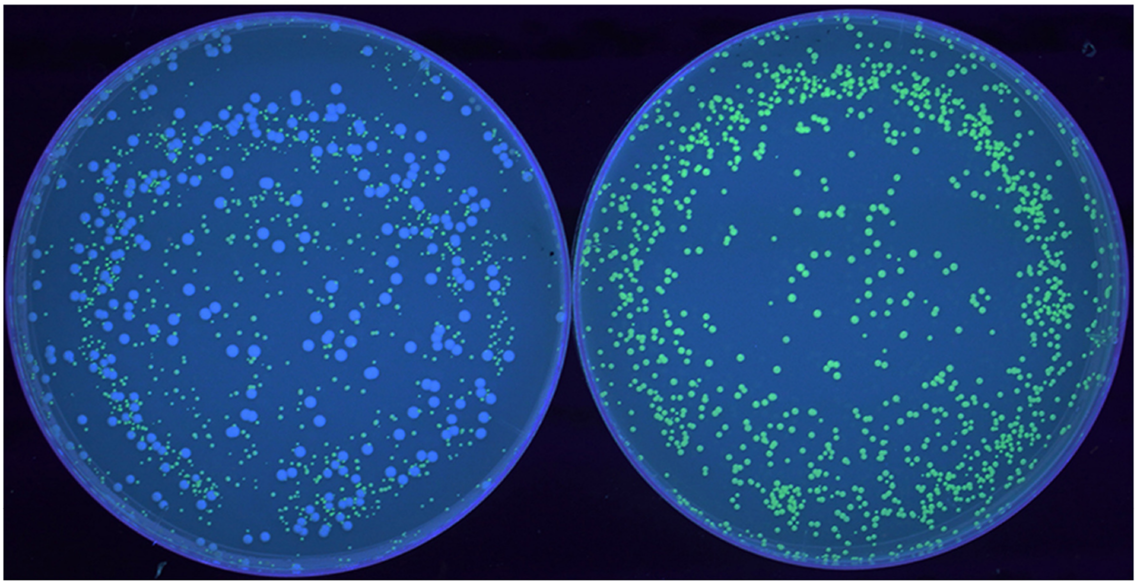

Figure S1. Reduction of adverse effect of the GFPmut3b expression by introducing Cycle3 mutations into GFPmut3b.

S. aureus RN4220 harboring pKF51 (GFPmut3b) (a) or pKF52 (GFP_{sa}) (b) were grown in TSB containing chloramphenicol at 37˚C overnight. The overnight culture dilutions (1:100,000) were plated onto TSB agar plates containing chloramphenicol and the plate were incubated for 24 h at 37˚C. After the plates were incubated at 37˚C for 24 h, the *S. aureus* colonies were photographed in UV light.

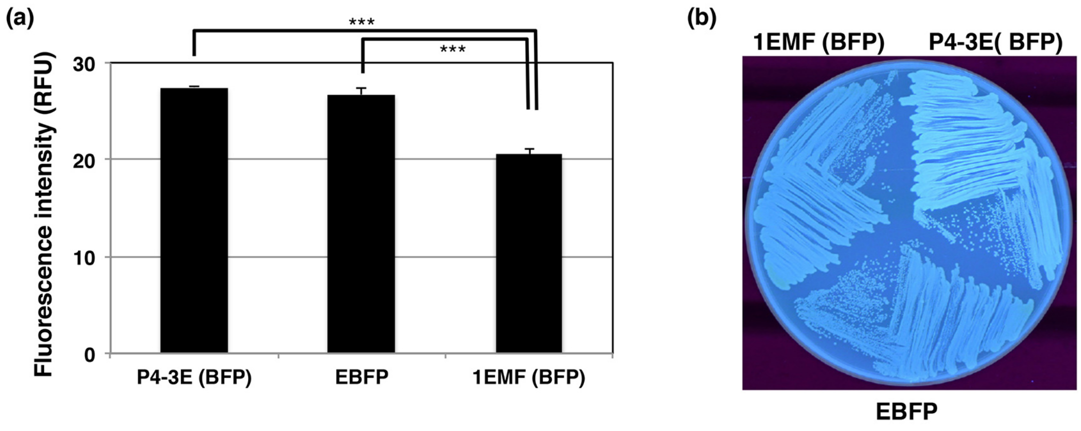

Figure S2. Comparison of fluorescence intensity among the three BFP variants.

(a) The fluorescent intensities of 4P-3E (BFP), EBFP, and 1EMF (BFP) in *S. aureus* MW2. The fluorescent intensities at 447 nm were measured with a microplate reader, λ_{ex} = 380 nm. The data represent mean values ± standard deviation. The statistical analysis was performed using Student's t-test. ***, $P < 0.001$ (b) The fluorescent colonies were photographed under UV excitation. *S. aureus* strain MW2 expressing 4P-3E (BFP), EBFP, and 1EMF (BFP) were grown on TSB agar plate containing chloramphenicol.

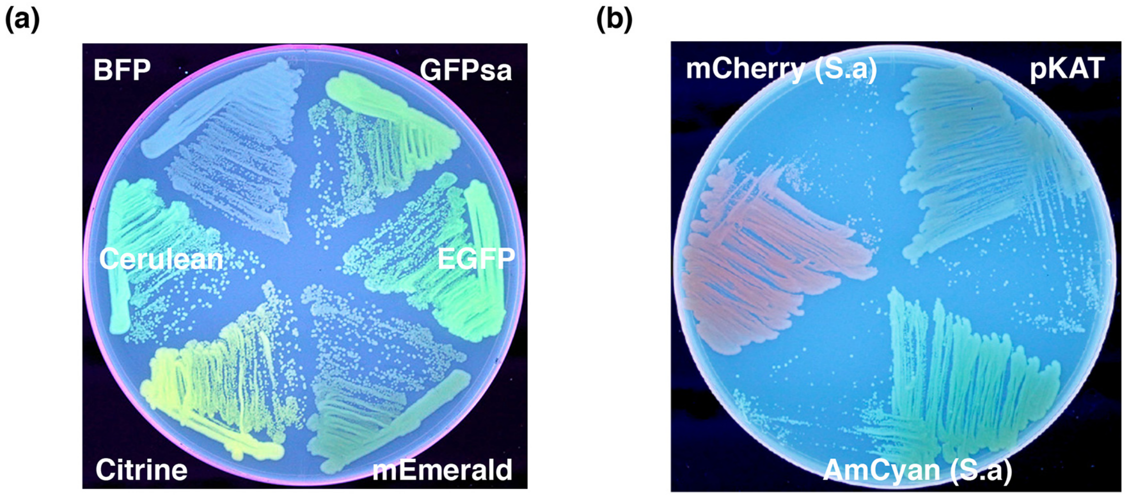

Figure S3. Expression of fluorescent proteins in the clinical strain N315.

The fluorescing colonies were photographed under UV excitation. (a) multicolor GFP variants (b) codon-optimized AmCyan and mCherry in *S. aureus* N315.

(a)

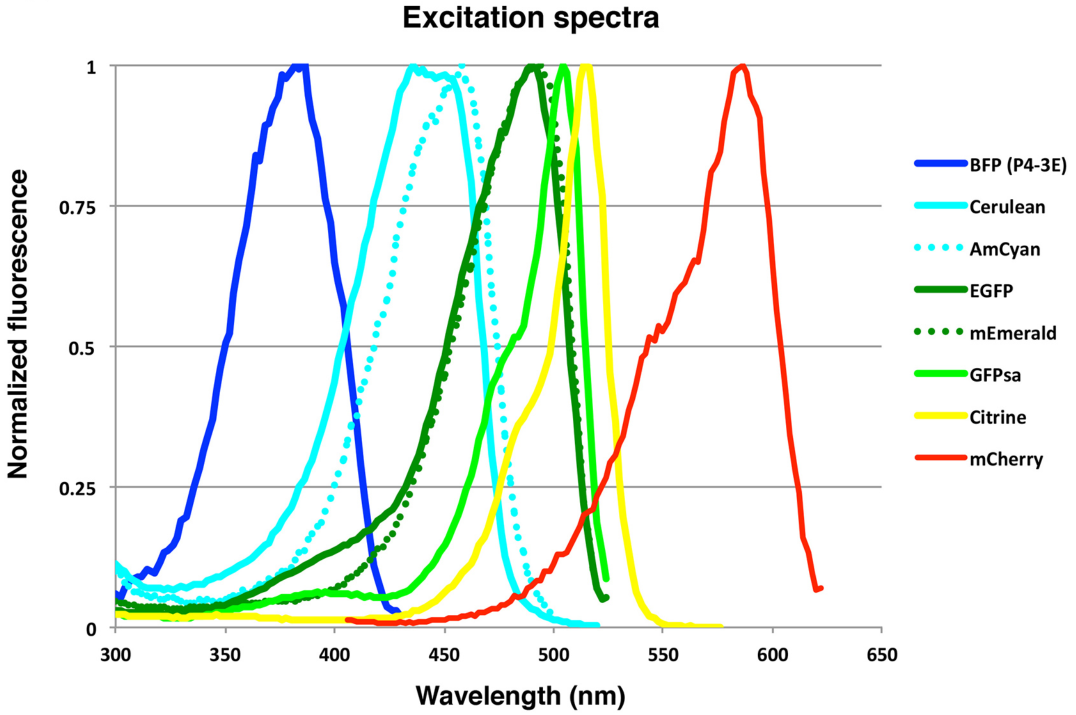
Excitation spectra
Normalized fluorescence
1
0.75
0.5
0.25
0
300
350
400
450
500
550
600
650
Wavelength (nm)
BFP (P4-3E)
Cerulean
AmCyan
EGFP
mEmerald
GFPsa
Citrine
mCherry

(b)

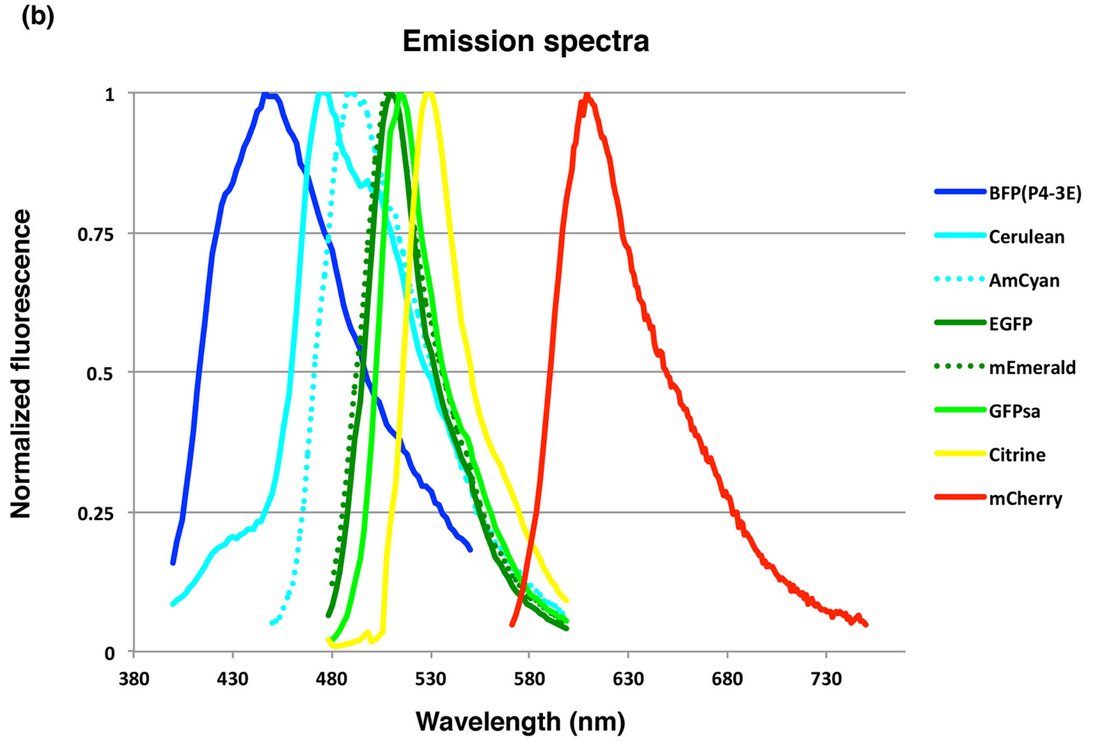
Emission spectra
Normalized fluorescence
1
0.75
0.5
0.25
0
380
430
480
530
580
630
680
730
Wavelength (nm)
BFP(P4-3E)
Cerulean
AmCyan
EGFP
mEmerald
GFPsa
Citrine
mCherry

Figure S4. Excitation and emission spectra for GFP_{sa}, EGFP, mEmerald, Citrine, Cerulean, BFP (P4-3E), mCherry, and AmCyan in *S. aureus.* Each spectrum was normalized to a maximum value of 1. (a) Excitation spectra were recorded with emission at 540 nm for GFP_{sa}, EGFP, mEmerald, and Citrine, 475 nm for Cerulean and BFP (P4-3E), 520 nm for AmCyan, and 640 nm for mCherry. (b) Emission spectra were recorded with excitation at 460 nm for GFP_{sa}, EGFP and Citrine, 380 nm for Cerulean and BFP (P4-3E), 430 nm for AmCyan, and 550 nm for mCherry.

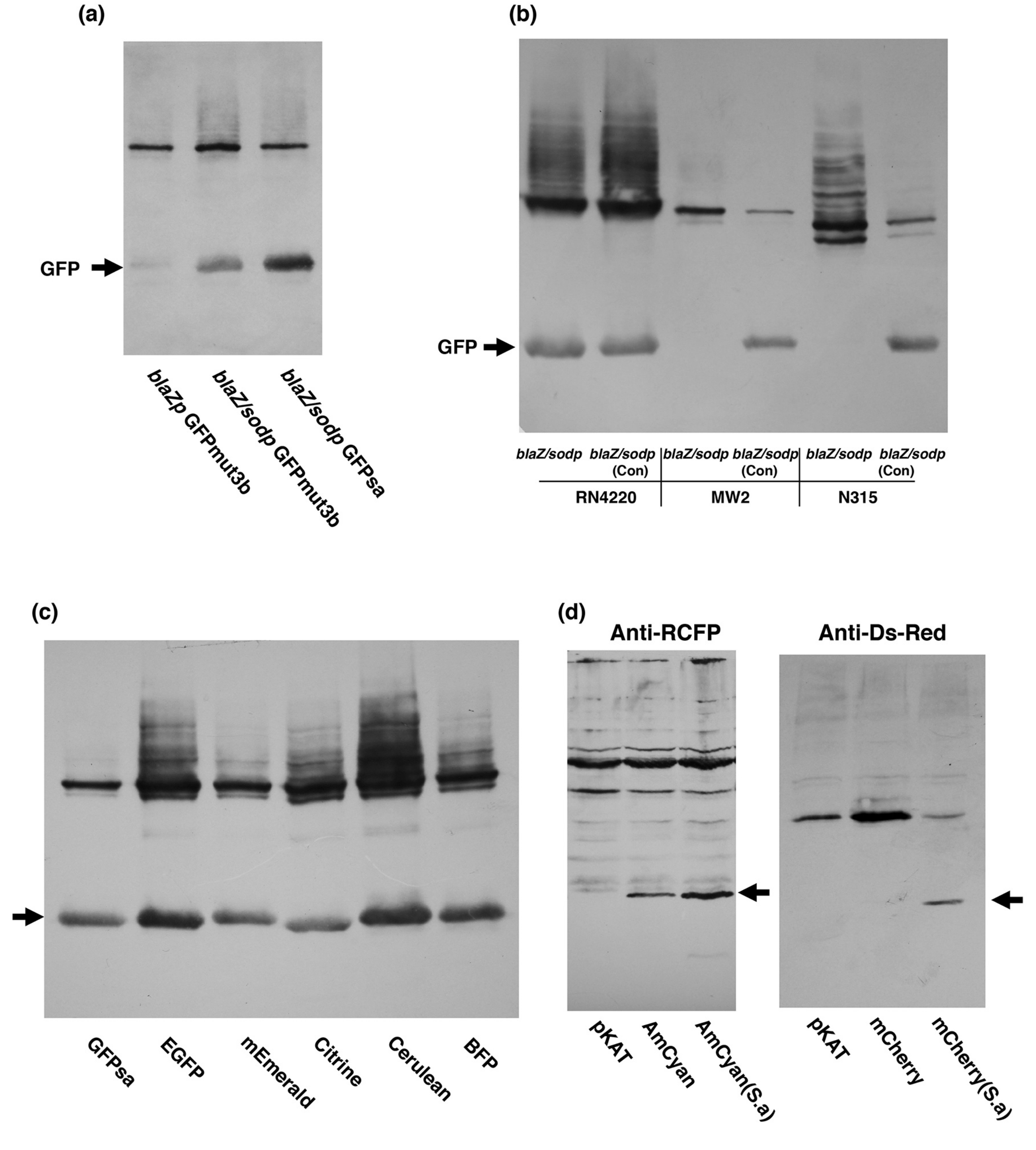

Figure S5

(a) The uncropped full-length dots of Fig. 1(d). (b) The uncropped full-length dots of Fig. 2(d).

(c) The uncropped full-length dots of Fig. 3(c). (d) The uncropped full-length dots of Fig. 4(c).
